# Analysis of Flavonoid in Medicinal Plant Extract Using Infrared Spectroscopy and Chemometrics

**DOI:** 10.1155/2016/4696803

**Published:** 2016-07-26

**Authors:** Lestyo Wulandari, Yuni Retnaningtyas, Nuri, Hilmia Lukman

**Affiliations:** Faculty of Pharmacy, University of Jember, Jember, East Java 68121, Indonesia

## Abstract

Infrared (IR) spectroscopy combined with chemometrics has been developed for simple analysis of flavonoid in the medicinal plant extract. Flavonoid was extracted from medicinal plant leaves by ultrasonication and maceration. IR spectra of selected medicinal plant extract were correlated with flavonoid content using chemometrics. The chemometric method used for calibration analysis was Partial Last Square (PLS) and the methods used for classification analysis were Linear Discriminant Analysis (LDA), Soft Independent Modelling of Class Analogies (SIMCA), and Support Vector Machines (SVM). In this study, the calibration of NIR model that showed best calibration with *R* ^2^ and RMSEC value was 0.9916499 and 2.1521897, respectively, while the accuracy of all classification models (LDA, SIMCA, and SVM) was 100%. *R* ^2^ and RMSEC of calibration of FTIR model were 0.8653689 and 8.8958149, respectively, while the accuracy of LDA, SIMCA, and SVM was 86.0%, 91.2%, and 77.3%, respectively. PLS and LDA of NIR models were further used to predict unknown flavonoid content in commercial samples. Using these models, the significance of flavonoid content that has been measured by NIR and UV-Vis spectrophotometry was evaluated with paired samples *t*-test. The flavonoid content that has been measured with both methods gave no significant difference.

## 1. Introduction

Indonesia shows an amazing diversity of plants species that have been associated with the human health from time immemorial. Many of them were reported to have various desirable activities; however, only 20–22% were cultivated [1]. Research of Indonesian medicinal plants using modern laboratory facilities has been started since 1970, but only about 200 plants were studied. This figure shows a very small portion of the overall number of medicinal plant species that were reported [2]. Therefore, the analysis of chemical constituents would help in determining various biological activities of plants.

Studies have shown that many plants have chemical components and biological activities. The most important of these bioactive constituents of plant are alkaloids, flavonoids, terpenoids, steroids, tannins, and saponins [3]. Flavonoids are the most common and widely distributed group of plant phenolic compounds, occurring virtually in all plant parts, particularly the photosynthesising plant cells [4]. Flavonoids have been reported to exert multiple biological effects, including antioxidant, free radical scavenging abilities, anti-inflammatory, and anticarcinogen [5].

Several analytical techniques have been developed for determining total flavonoids concentration such as gas chromatographic (GC) [6], mass spectrometry [7], thin layer chromatography [8], UV spectrometry [9], and high performance liquid chromatography (HPLC) [10]. These methods are precise but all time-consuming, requiring many reagents, and costly [11]. Therefore, a simple, selective, and ecofriendly method is required.

Infrared spectroscopy is a technique based on the vibrations of the atoms of a molecule. The advantage of the infrared technique is that it can be nondestructive, requires a relatively small amount of sample, is fast, and is accurate [11, 12]. Infrared technique does not require a reagent, so this method is more ecofriendly. It has been proved to be a powerful analytical tool used in many fields [12]. In recent years, NIR combined with chemometrics has attracted considerable attention in chemical content analysis [13]. NIR spectroscopy also shows promising ability for discrimination of similar biological materials, such as pea [14], fruits [15], and wine [16]. Some papers have been published regarding NIR quantitative analysis of active compound concentration in herbal products [17].

Multivariate statistical methods are very useful for processing of IR spectra. The big advantage of multivariate statistical methods is their capability to extract the information of IR spectra and explore this spectral information for qualitative or quantitative applications. The most frequently used of multivariate statistical methods (often called chemometric methods) are Linear Discriminant Analysis (LDA) and Partial Least Squares (PLS) regression [18].

The objective of this research is to develop a simple, rapid, and validated model of IR spectra for the determination of the flavonoid content. Furthermore, IR spectroscopy and chemometric methods were applied for determining flavonoid content in commercial samples.

## 2. Materials and Methods

### 2.1. Material and Reagents

In this study, samples used were leaves samples collected from Materia Medica Botanical Garden, Malang, Indonesia (Table 1). Methanol, ethanol, Folin-Ciocalteu, potassium acetate (E. Merck, Darmstadt, Germany), and quercetin (Sigma-Aldrich) were of analytical grade reagent. Aquadest and Aerosil were of pharmaceutical grade. The solvents were used without further purification. Commercial extract capsules, Stimuno® and Daun Salam®, were purchased from a local pharmacy in Jember, East Java, Indonesia (October 2015).

### 2.2. Extraction Methods

Dry leaves samples were mixed and finely powdered. 80.0 g of powdered sample was extracted with 800 mL of methanol in an ultrasonicator for an hour and continued being extracted by maceration for 24 hours. The extract was filtered through Whatman filter paper and then the solvent was evaporated using a rotavapour at 60°C. Extract was dried using Aerosil to yield dry extract.

### 2.3. NIR Spectra Acquisition

Samples were scanned with a Brimrose, Luminar 3070 (Brimrose Corp, Baltimore, MD), with an integrating sphere. Before samples were measured, the instrument was warmed up for 30 minutes. The monochromator entrance slit was set on 500 pm, the amplifier was set on 200. the response time is smooth (1 ms), and light intensity was set on 14 volts. The wavelength range of spectra is from 8500–2000 nm and the data were measured in 5 nm intervals, which resulted in 120 points reflection.

### 2.4. FTIR Spectra Acquisition

FTIR spectrometer (Alpha FTIR Spectrometer from Bruker optic), equipped with a deuterated triglycine sulphate (DTGS) as a detector and a germanium as beam splitter, interfaced to computer operating under Windows-based system, and connected to software of OPUS operating system (Version 7.0 Bruker optic), was used during FTIR spectra acquisition. A few drops of each sample were positioned in contact with attenuated total reflectance (ATR) plate.

FTIR spectra were collected at frequency regions of 4000–650 cm^−1^ by coadding 32 scans and at resolution of 4 cm^−1^. All spectra were substracted against a background of air spectra. After every scan, a new reference of air background spectra was taken. The ATR plate was carefully cleaned by scrubbing with isopropyl 70% twice followed by drying with soft tissue before being filled in with the next sample, making it possible to dry the ATR plate. These spectra were recorded as absorbance values at each data point in replicate two times.

### 2.5. Determination of Total Flavonoids Content

The flavonoids content was determined by aluminum chloride method using quercetin as a reference compound [19]. Sample was prepared by mixing 0.5 mL of 4 mg/mL sample extract in ethanol with 3 mL of ethanol, 0.2 mL of 10% aluminum chloride, and 0.2 mL of 1 M potassium acetate and then diluted to 25 mL with distilled water. After incubation at room temperature for 30 min, the absorbance of the mixture solution was measured at 432 nm using spectrophotometer (UV-Vis Hitachi U 1800). Various standard solutions of quercetin (2.0 up to 15.0 *μ*g/mL) were prepared from two stock solutions by dilution with ethanol.

### 2.6. Calibration and Classification Models

Chemometric analysis was performed using The Unscrambler software package (Version 10.2. CAMO ASA, Norway). Calibration and classification model for determination of flavonoids content were formed by training set samples that consists of fifteen medicinal plant extracts, quercetin, Aquadest, and Aerosil. The training set samples of medicinal plant extracts have been traced having varied flavonoid content which is expected to represent variations of flavonoid content of all plants. The Linear Discriminant Analysis (LDA), Soft Independent Modelling of Class Analogies (SIMCA), and Support Vector Machines (SVM) were used to develop classification model. These models were using two kinds of category, matrix and flavonoid. Matrix category was sample without flavonoids content (Aquadest and Aerosil) and flavonoid category was sample with flavonoid content (leaves extracts and quercetin). Partial Least Square (PLS) was used to develop calibration model for total flavonoids content. The PLS model was then validated with leave-one-out cross-validation (LOOCV) and 2-fold cross-validation (five test set samples). The training set and test set samples were shown in Table 2.

## 3. Result and Discussion

### 3.1. Total Flavonoids Content

The results for total flavonoids content in samples are presented in Table 3. The total flavonoids measurements were distributed around 4.03 up to 51.49 mg quercetin equivalence (QE)/g extract.

### 3.2. Calibration and Classification Models

#### 3.2.1. Calibration and Classification of NIR Models

**Figure 1:**
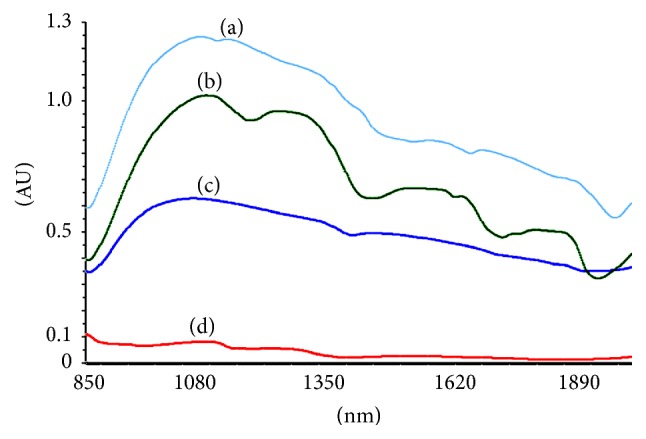
NIR spectra of quercetin (a), dry extract (b), Aquadest (c), and Aerosil (d).

**Figure 2:**
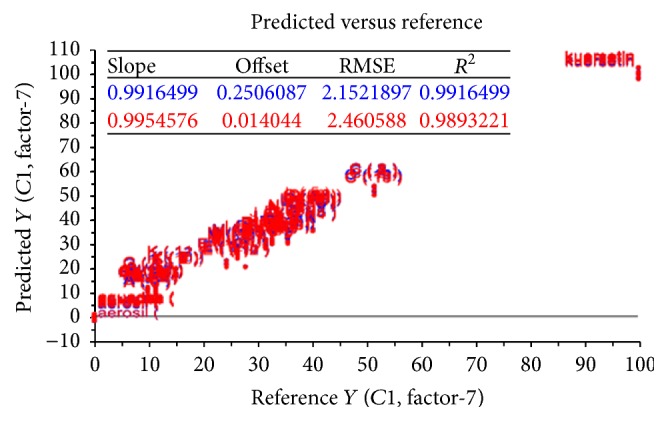
The correlation data of PLS (NIR model).

Figure 1 showed NIR spectra of quercetin, dry extract, Aquadest, and Aerosil. Those spectra have a different intensity and typical characteristic of absorption bands. In the PLS calibration models, the evaluation of the linearity method was carried out in order to show a proportional relationship between the absorbance of NIR spectra versus the concentrations of flavonoid. The absorbance data of all training set were obtained at 850–2000 nm. The correlation data of PLS model in Figure 2 showed good performance of PLS model, indicated by coefficient of determination (*R* ^2^) higher than 0.99 and the low value of RMSEC [20]. *R* ^2^ and the root mean square error of calibration (RMSEC) were 0.9916499 and 2.1521897, respectively. Therefore the calibration model can be used as a tool to predict the concentration of flavonoid content in medicinal plant.

**Figure 3:**
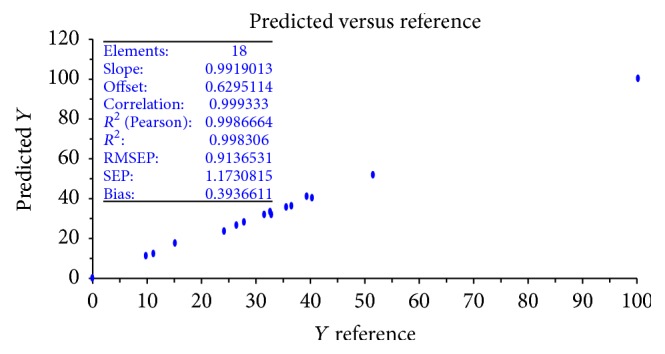
The leave-one-out cross-validation (LOOCV) of PLS (NIR model).

In order to validate the developed model, leave-one-out cross-validation (LOOCV) and 2-fold cross-validation were used. LOOCV was performed as follows: one sample was left out from the calibration set, a model was built with the remaining samples in the calibration set, then the left-out sample was predicted by this model, and the procedure was repeated by leaving out each sample in the calibration set. *R* ^2^ and the root mean square error of prediction (RMSEP) of LOOCV were 0.9986664 and 0.9136531, respectively (Figure 3).

**Figure 4:**
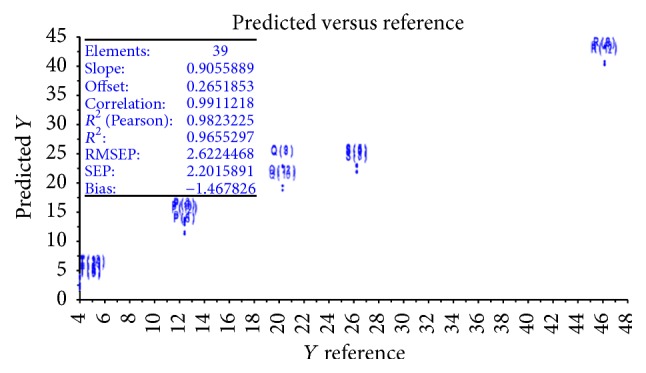
2-fold cross-validation of PLS (NIR model).

Twofold cross-validation was used to validate the developed model using independent samples (test set). Five medicinal plant extracts were used as test set. *R* ^2^ and RMSEP were 0.9823225 and 2.6224468, respectively (Figure 4).

The ability of NIR model (LDA, SIMCA, and SVM) to classify samples in flavonoid and matrix category can be seen through the accuracy of classification models.  Table 4 shows 100% of accuracy, which means that the model could classify fifteen training set samples in a correct category.

#### 3.2.2. Calibration and Classification of FTIR Models

**Figure 5:**
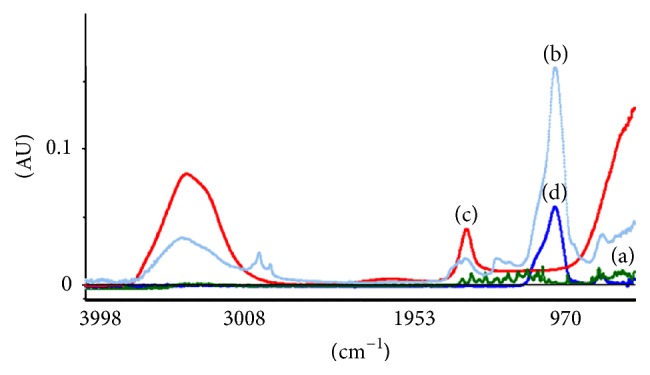
FTIR spectra of quercetin (a), dry extract (b), Aquadest (c), and Aerosil (d).

Figure 5 showed FTIR spectra of quercetin, dry extract, Aquadest, and Aerosil. Those spectra have similar intensity and characteristic of absorption bands in some segment of wavenumber. The results obtained from the PLS in terms of *R* ^2^, RMSEC for normal spectra, and segmentation were presented in Table 5. PLS calibrations in segment C (1650–1400 cm^−1^) revealed the highest of *R* ^2^ and the lowest of RMSEC compared with other segments (Table 5). *R* ^2^ and RMSEC were 0.8653689 and 8.8958149, respectively. However, this result was not good due to the fact that *R* ^2^ was less than 0.99 and RMSEC was high.

The ability of FTIR model (LDA, SIMCA, and SVM) was less than 100%, which means that the model could not classify fifteen training set samples in a correct category (Table 6).

### 3.3. Application of Commercial Samples

PLS and LDA developed models of NIR spectra were further used to predict flavonoid in commercial samples. The results of flavonoids content in samples measured by NIR and UV-Vis spectrophotometry method are presented in Table 7. The paired samples* t*-test shows that flavonoid content that has been measured with both methods gave no significant difference (*p* > 0.05). Furthermore, all of the commercial samples were in the flavonoid category in LDA model.

## 4. Conclusion

The NIR spectroscopy combined with multivariate calibrations methods can be used to determine flavonoid in medicinal plant extract. The suggested method is simple, selective, validated, and ecofriendly.

## Figures and Tables

**Table 1:** Identity code of samples.

Number	Code	Leaves samples
(1)	A	*Coffea arabica *(young)
(2)	B	*Coffea arabica *(old)
(3)	C	*Psidium guajava*
(4)	D	*Sauropus androgynus*
(5)	E	*Tithonia diversifolia*
(6)	F	*Mangifera indica*
(7)	G	*Pandanus amaryllifolius*
(8)	H	*Momordica charantia*
(9)	I	*Euphorbiae hirtae*
(10)	J	*Carica papaya*
(11)	K	*Mimosa pudica*
(12)	L	*Andrographis paniculata*
(13)	M	*Piper ornatum*
(14)	N	*Piper betle*
(15)	O	*Annona muricata*
(16)	P	*Anredera cordifolia*
(17)	Q	*Kaempferia rotunda*
(18)	R	*Leucaena glauca*
(19)	S	*Morinda citrifolia*
(20)	T	*Coffea canephora *(old)

**Table 2:** Training set and test set samples.

Number	Samples code	Identity of group
(1)	A	Training set
(2)	B	Training set
(3)	C	Training set
(4)	D	Training set
(5)	E	Training set
(6)	F	Training set
(7)	G	Training set
(8)	H	Training set
(9)	I	Training set
(10)	J	Training set
(11)	K	Training set
(12)	L	Training set
(13)	M	Training set
(14)	N	Training set
(15)	O	Training set
(16)	P	Test set
(17)	Q	Test set
(18)	R	Test set
(19)	S	Test set
(20)	T	Test set

**Table 3:** Total flavonoids content in samples.

Number	Samples code	mg QE/g extract ± SD
(1)	A	9.87 ± 0.25
(2)	B	11.23 ± 0.39
(3)	C	51.49 ± 0.21
(4)	D	31.53 ± 0.02
(5)	E	24.17 ± 0.10
(6)	F	35.61 ± 0.01
(7)	G	9.74 ± 0.48
(8)	H	32.74 ± 0.87
(9)	I	27.87 ± 0.02
(10)	J	32.55 ± 0.07
(11)	K	15.06 ± 0.13
(12)	L	39.22 ± 0.11
(13)	M	26.41 ± 0.17
(14)	N	36.46 ± 0.04
(15)	O	40.25 ± 0.25
(16)	P	14.39 ± 0.09
(17)	Q	20.25 ± 0.72
(18)	R	46.07 ± 0.28
(19)	S	26.23 ± 0.78
(20)	T	4.03 ± 0.07

**Table 4:** The accuracy of classification of NIR model (LDA, SIMCA, and SVM).

Model	Accuracy
LDA	100%
SIMCA	100%
SVM	100%

**Table 5:** The calibration of FTIR model.

Wavelength number	*R* ^*2*^ calibration	*R* ^*2*^ validation	RMSEC	RMSECV
4000–500 cm^−1^	0.8558883	0.5403671	9.2037029	16.860432
3500–3000 cm^−1^	0.8527114	0.5758341	11.093782	18.985434
1300–1000 cm^−1^	0.8234164	0.7321395	10.187981	12.915498
1650–1400 cm^−1^	0.8653689	0.8201284	8.8958149	10.315225

**Table 6:** The accuracy of classification of FTIR model (LDA, SIMCA, and SVM).

Model	Accuracy
LDA	86.0%
SIMCA	91.2%
SVM	77.3%

**Table 7:** Analysis of flavonoid content with NIR and UV-Vis spectrophotometry.

Commercial sample	Flavonoid content (mg QE/g extract) with NIR	Flavonoid content (mg QE/g extract) with UV-Vis spectrophotometry
Stimuno	36.30 ± 2.78^*∗*^	35.94 ± 0.14^*∗*^
Daun Salam	17.18 ± 0.06^*∗*^	15.12 ± 0.02^*∗*^

^*∗*^SD (*n* = 3).
